# Evaluation of metabolic profile and renal function of treatment of Duguetia furfuracea aqueous extract in obese female Wistar rats

**DOI:** 10.1186/1758-5996-7-S1-A126

**Published:** 2015-11-11

**Authors:** Dionys de Souza Almeida, Dener Lucas Araújo dos Santos, Aline Zanatta Schavinski, Amanda Lima Deluque, Eduardo Kloppel, Carolina Abreu Miranda, Kleber Eduardo de Campos

**Affiliations:** 1 Universidade Federal de Mato Grosso, Barra do Garças, Brazil

## Background

Obesity is a pathophysiology that influences the metabolism, worsening the lipidic and glycemic profiles, and the population search for alternatives to decrease its side effects. Duguetia furfuracea has been used for obesity and also kidney disease treatment, however a very few studies have been conducted to verify its effectiveness and safety for using as well as to evaluate its influence on metabolic profile.

## Objective

To evaluate the effects of aqueous extract of D. furfuracea in metabolic profile and renal function in obese Wistar female rats.

## Materials and methods

D. furfuracea leaves were dried and proceeded to the preparation of the extract by infusion. Twenty newborn female Wistar rats were used, were administrated monosodium glutamate solution, 4.0 mg/Kg body weight (obese) in neonatal period. At adult age (120 days of life), they were into two groups: control (CONT; n=10) daily treated with water; and experimental (DF n=10) daily treated with aqueous extract of D. furfuracea at a dose of 200 mg/Kg. The treatment length for 28 days. Lee Index for obesity classification was measured in first and last day of experimental protocol, and weekly it was measured body weight, blood glucose, urine flow, fecal weight, and food and water consumption. At 28th day of treatment, all rats were anesthetized and killed by decapitation and thus serum biomarkers were measured [total protein, cholesterol and its fractions (HDL, LDL and VLDL), triglycerides and liver transaminases (ALT and AST)]. Moreover, several organs were collected (liver, kidney and periovarian fat) and also performed their relative weight. Statistical significance was p <0.05.

## Results

Obesity was confirmed by Lee index, showing higher values of 0.300, demonstrating the success of obesity induction method. Food intake was increased on days 14 and 21 in CONT group, and only the day 14 in DF group, all compared to the first day of treatment (day 0). The glycemic values in CONT rats were lowered at days 7 and 21, compared to day 0 compared to day zero (0). Water intake, fecal weight, urine flow and biochemical data did not present any alterations.

## Conclusion

The dose used in treatment of D. furfuracea in experimental obesity did not change the metabolic profile, nor presented alteration in renal function.

